# Climate Change and the Distribution of Neotropical Red-Bellied Toads (*Melanophryniscus*, Anura, Amphibia): How to Prioritize Species and Populations?

**DOI:** 10.1371/journal.pone.0094625

**Published:** 2014-04-22

**Authors:** Caroline Zank, Fernando Gertum Becker, Michelle Abadie, Diego Baldo, Raúl Maneyro, Márcio Borges-Martins

**Affiliations:** 1 Programa de Pós-graduação em Biologia Animal, Departamento de Zoologia, Instituto de Biociências, Universidade Federal do Rio Grande do Sul, Porto Alegre, RS, Brazil; 2 Departamento de Ecologia, Instituto de Biociências, Universidade Federal do Rio Grande do Sul, Porto Alegre, RS, Brazil; 3 Instituto de Biología Subtropical (CONICET-UNaM), Laboratorio de Genética Evolutiva, Facultad de Ciencias Exactas, Químicas y Naturales, Universidad Nacional de Misiones, Posadas, Misiones, Argentina; 4 Laboratorio de Sistemática e Historia Natural de Vertebrados, Facultad de Ciencias, Universidad de la República, Montevideo, Uruguay; Cirad, France

## Abstract

We used species distribution modeling to investigate the potential effects of climate change on 24 species of Neotropical anurans of the genus *Melanophryniscus*. These toads are small, have limited mobility, and a high percentage are endangered or present restricted geographical distributions. We looked at the changes in the size of suitable climatic regions and in the numbers of known occurrence sites within the distribution limits of all species. We used the MaxEnt algorithm to project current and future suitable climatic areas (a consensus of IPCC scenarios A2a and B2a for 2020 and 2080) for each species. 40% of the species may lose over 50% of their potential distribution area by 2080, whereas 28% of species may lose less than 10%. Four species had over 40% of the currently known occurrence sites outside the predicted 2080 areas. The effect of climate change (decrease in climatic suitable areas) did not differ according to the present distribution area, major habitat type or phylogenetic group of the studied species. We used the estimated decrease in specific suitable climatic range to set a conservation priority rank for *Melanophryniscus* species. Four species were set to high conservation priority: *M. montevidensis*, (100% of its original suitable range and all known occurrence points potentially lost by 2080), *M*. sp.2, *M. cambaraensis*, and *M. tumifrons*. Three species (*M. spectabilis*, *M. stelzneri*, and *M*. sp.3) were set between high to intermediate priority (more than 60% decrease in area predicted by 2080); nine species were ranked as intermediate priority, while eight species were ranked as low conservation priority. We suggest that monitoring and conservation actions should be focused primarily on those species and populations that are likely to lose the largest area of suitable climate and the largest number of known populations in the short-term.

## Introduction

Projections of future climate predict that major changes will take place in most subtropical regions, including an increase in average global temperature and a decrease in precipitation [1], [2]. Current global climate distribution is likely to change during the 21st century, and possibly some climate patterns will disappear and others will emerge [3]. Impacts of global climate change can already be observed in several physical and biological systems [4], [5], [6], and these impacts might change the distribution of suitable areas for a wide variety of organisms by the end of the century, increasing the risk of extinction for many species [7], [8], [9], particularly those with restricted geographical range [10], [11]. In addition, species that are already threatened might suffer further negative changes in conservation status [12].

Population persistence under climate change is dependent either on adaptation or dispersal capabilities that enable species to track suitable habitat conditions in other areas [13], [14]. Species that do not display either of these abilities will probably become extinct [15], so that biodiversity may decline and highly mobile and opportunistic species may thrive [16], [17]. Amphibians are experiencing accelerating worldwide population declines and species extinctions [18]. Approximately 30% of all amphibian species are currently listed as threatened to some degree by the International Union for Conservation of Nature [19]. Amphibian decline is considered a global problem with complex local causes rooted in climate change, and in habitat alteration and fragmentation caused by human activities [8], [18], [20], [21]. Amphibians are particularly vulnerable to climate change given their aquatic/terrestrial life histories and low dispersal abilities. Therefore, displacement and contraction of suitable climatic areas represents a major threat to the conservation of amphibian species with low dispersal capabilities [22], [23]. The potential effects of climate change can be evaluated by developing models that provide working hypotheses to support research and conservation strategies. In the particular case of Neotropical amphibians, it is crucial to prioritize species and populations for *in situ* monitoring, with the aim of supporting conservation decisions, but also for evaluating whether the real effects correspond to those projected by models.

In this study, we used species distribution modeling (SDM, *sensu* [24]) to investigate the potential effects of climate change projected for 2020 and 2080, for 24 species of anurans of the Neotropical genus *Melanophryniscus*. These toads are small in size (less than 40 mm), and limited in mobility, and a high percentage of species are endangered and/or restricted in their geographical distribution [19]. To compile the species data, we gathered a comprehensive taxonomic and georreferenced list of 4,000 records from 22 scientific collections in six countries. Our main objective was to estimate the potential effects of climate change on the distribution of *Melanophryniscus* species and to use the results for prioritizing species for conservation. Based on comparisons of present time modeled distributions and distributions projected to the future, we aimed to answer the following questions: (1) What is the potential magnitude of change in the size of suitable climatic area for each species?; (2) What is the potential number of currently known populations of each species that would be located in non-suitable climatic conditions in the future (i.e., the potential loss of known populations)?; (3) Is the reduction in suitable areas related to Major Habitat Type, original range size or phylogeny (inferred from species groups); and (4) Can we rank species and populations for prioritization in research and conservation?

## Material and Methods

### Study species

**Figure 1:**
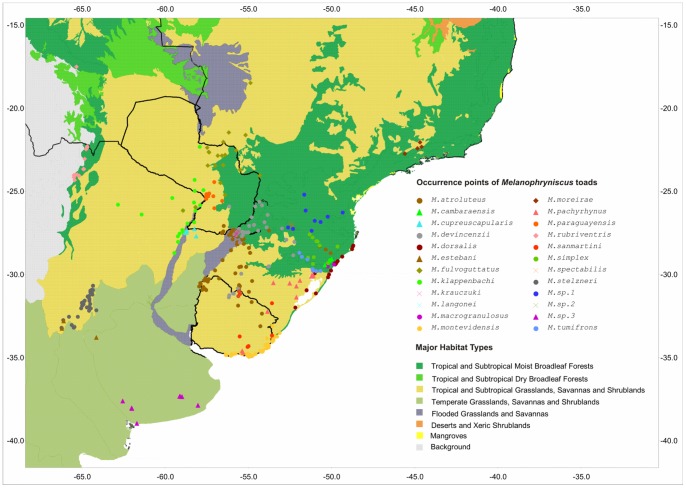
Occurrence records of *Melanophryniscus*. Points in the map represent the known distribution of the studied *Melanophryniscus* species throughout the major habitat types of South America.

*Melanophryniscus* Gallardo, 1961, is a Neotropical genus that was recovered as the sister taxon of all remaining Bufonidae in several phylogenetic analyses (e.g. [25], [26], [27]). The distribution of this genus is restricted to subtropical and tropical South America, including northern Argentina, central and southern Brazil, Uruguay, Paraguay, and Bolivia (Fig. 1). *Melanophryniscus* is currently represented by 26 recognized species [28], [29], [30], [31]. The majority of these species exhibit naturally small distributions, and in some cases, the distribution of a species is limited to only one or two known locations. It is also remarkable that their distributions are mostly non-sympatric (Fig. 1). In addition, these toads are considered rare and/or difficult to record. They are normally difficult to find during most of the year and are usually recorded only during explosive reproductive events (sensu [32]), which occur over a short period of time in temporary aquatic environments created during- and immediately after intense rainfall [33], [34], [35]. Precipitation and temperature appear to strongly influence the activity patterns of the species in this genus (e.g. [33], [35]). *Melanophryniscus* is a group under strong conservation concern, with at least 10 species included on endangered species lists at regional, national or global levels and three others listed as Data Deficient [19], [36], [37], [38], [39], [40]. This genus therefore includes a group of vulnerable species that might experience shifts of climatic suitability throughout their actual distribution range.

We used data from 24 species (Dataset S1), including 21 recognized species in the genus *Melanophryniscus* [28], [30] and three undescribed species, hereafter referred to as *M*. sp.1, *M*. sp.2, and *M*. sp.3. Five recently described species, *M. admirabilis* Di-Bernardo, Maneyro, and Grillo, 2006; *M. alipioi* Langone, Segalla, Bornschein, and de Sá, 2008; *M. vilavelhensis* Steinbach-Padilha, 2008; *M. peritus* Caramaschi and Cruz, 2011; and *M. setiba* Peloso, Faivovich, Grant, Gasparini and Haddad, 2012, were excluded from the analyses because they are each known from one single locality.

Presence localities were obtained primarily from the published literature (Dataset S2) and later validated by a review of voucher specimens. We reviewed approximately 4,000 voucher specimens deposited in 22 scientific collections in Argentina, Brazil, Uruguay, Paraguay, Germany, and France (Dataset S3), including all historical records. To determine the geographical coordinates for each record of species presence, we used data available in the scientific literature or from voucher specimens in museum collections. When the precise geographic locations were not available, we assigned approximate coordinates according to descriptions of sample localities as they appear in museum records and in the literature, or to the closest town. We checked all locations using the on-line tools available in the SpeciesLink project (http://splink.cria.org.br/)[41].

### Environmental data

For each species, we generated models of current suitable areas using altitude and nine bioclimatic variables obtained from the WorldClim version 1.4 database (Dataset S4), which is based on the interpolation of climatic conditions recorded from 1950 to 2000, with a 30 s (≈1 km) resolution [42]. We selected these ten variables because they have low collinearity [43], and also because they are ecologically meaningful to amphibians: mean diurnal range, isothermality, maximum temperature of warmest month, annual temperature range, mean temperature of wettest quarter, mean temperature of the warmest quarter, precipitation of the wettest month, precipitation seasonality, precipitation of the driest quarter and altitude.

To assess the effects of climate change, we used climatic data projected to the years 2020 and 2080, from the global climate model of the Canadian Centre for Climate Modeling and Analysis (CCCMA), which was recently evaluated as a top performing model [44]. In addition, we used data specified by emission scenarios (SRES) A2a and B2a described in the 2001 Intergovernmental Panel on Climate Change (IPCC) Third Assessment Report, which simulated climate system responses to increasing levels of greenhouse gases based on different hypotheses about projected population size, technological advances and socioeconomic trends. Scenario A2a projects relatively large changes based on recent observations suggesting that climate change will be more severe than previously expected [5], [45], [46]. Scenario B2a projects intermediate climate changes, based on projections of a world with intermediate population and economic growth [5]. Projected data for the maximum temperature, minimum temperature and average precipitation for the years 2020 and 2080 were obtained from the WorldClim database and processed in the software DIVA-GIS, version 5.2 [47], to generate the same nine bioclimatic variables used for modeling the current distributions.

### Species distribution models

We generated species distribution models using the MaxEnt algorithm (MaxEnt version 3.3.3k), which searches for the maximum entropy density using Robust Bayes Estimation and requires only presence points as input data [48], [49], [50]. Ultimately, MaxEnt estimates the relation between species presence and environmental variables in a particular geographic space and draws a model of environmental suitability for the occurrence of a given organism. We estimated the current distribution of climatic suitability for *Melanophryniscus* species, projected the models on future climatic conditions and then compared the current and future distribution of climatic suitability.

We ran the MaxEnt software using the default settings, which have been validated in studies involving a variety of species and types of environmental data [50]. To obtain binary models of presence/absence from the continual logistic probabilities generated by MaxEnt, we selected a threshold at which the training sensitivity and specificity values were the same, minimizing the absolute difference between sensitivity and specificity [51]. This threshold approach yields low rates of both false positives and false negatives (both <0.2; [52]).

We restricted the geographical extent of the models and of the background sampling to a region between 0° and 56° degrees in latitude, and 34° and 81° degrees in longitude, which includes most of tropical and subtropical South America east of the Andes and covers the entire distribution of the genus in South America.

To evaluate model performance for each species, we used AUC values (Area Under the *Receiver Operating Characteristics* Curve) calculated through 10-fold cross-validation [49]. AUC values range from 0.5 for models with no predictive power to 1.0 for models with perfect predictive power [53]. AUC values greater than 0.9 denote “*very good*” predictive power, values between 0.8 and 0.9 denote “*good*” predictive power and values between 0.7 and 0.8 indicate “*useful*” predictive power [53]. Although AUC has known limitations as a measure of model performance [54], it still is the most used metric.

We produced maps of current and future suitable climatic areas for each of the 24 selected species of *Melanophryniscus*, and projected models of the future in different years (2020 and 2080) to take into consideration the short-, and long-term effects in two different scenarios of climate change.

### Potential effects on species and populations and ranking criteria

To estimate the effects of climate change on different species and populations, we used two different approaches. We evaluated the relative changes in their potential distribution areas and the relative changes in the probabilities of occurrence at each known presence location.

To evaluate the changes in the suitable climatic areas for each of the 24 species, we overlaid the current, 2020 and 2080 potential presence maps to check for coincident presence regions. These coincident regions are indicative of persistent presence from the present time to 2080. This overlay operation is algebraically equivalent to P_2080_ = P_present_*P_2020_*P_2080_; where P can be either 0 (absence) or 1 (presence). When P_2080_ = 1 for a given location, we then interpreted the predicted presence as persistent presence from the present time to 2080. This is a rigid estimation of potential persistence because it does not allow for recolonization of areas after a local extinction has taken place, even if climatic conditions become suitable again in the future. For example, if absence has been predicted for a given location in 2020, then Persistence_2080_ = 1*0*1, so that P_2080_ = 0 (i.e., 0 = predicted absence, or “local extinction” in 2080). The opposite result (and interpretation) would be obtained if two dates only had been considered (present date and 2080). In this case, Persistence_2080_ = 1*1 = 1 (i.e., 1 = predicted presence, or “long-term persistence from the present to 2080”). These analyses were based on the assumption that none of the studied species would be able to disperse to new potential areas within the modeling time frame, and therefore, any future increase in area was considered as not ecologically possible. We also assumed that no *Melanophryniscus* species would be able to adapt to new conditions. We made these assumptions due to the lack of information about the adaptive potential of *Melanophryniscus* species and because they seem to have low dispersal ability [35]. We acknowledge the debatable nature of these assumptions [24], [55], [56], however they can be considered plausible within the relatively short time frame (80 years) and spatial extent of our study, and considering that *Melanophryniscus* species are similar in size.

To quantify the effects of climate change at each known occurrence point, we used current and future (2020 and 2080) probabilities of occurrence generated by MaxEnt in a 5 km^2^ area centered on each point, using Idrisi Taiga GIS software [57]. This approach was used to attenuate the potential errors associated to using the values of a single point location (*i.e*., the unknown error in accuracy of the geographical coordinates of presence data taken from museum records). We assumed each presence location as a distinct population. For each species, we checked whether the probability of occurrence at each recorded presence point was above or below the presence threshold. Populations were then classified into two groups: (a) populations with occurrence probabilities below the presence threshold in 2020 and (b) populations with occurrence probabilities above the presence threshold in 2080.

We also evaluated whether differences in the magnitudes of area losses by 2080 were related to phylogeny (inferred from species groups), to the size of the original distribution range, and to Major Habitat Types (Dataset S5).

To prioritize the species according to the degree of impact caused by climate change, we used a scatterplot between the percent decreases in the distribution areas projected for each species by 2080 and the percent of occurrence points lost by 2080. Thus, species with both high percent loss in area by 2080 and high percent loss in known occurrence points should receive higher priority, while species with high percent decrease only in either area or known occurrence points would receive intermediate priority. Species with low decrease in both parameters would receive the lowest priority.

## Results

The models of suitable climatic areas presented high AUC values ranging from 0.96 to 1.00 (Table 1). The presence threshold values varied across species from less lenient thresholds, such as that observed for *Melanophryniscus cambaraensis* (threshold = 0.725), to more lenient values, such as that of *M. dorsalis* (threshold = 0.026) (Table 1).

**Table 1:** Summary of models.

Species (n)	AUC mean	Threshold	ALT	BIO 2	BIO 3	BIO 5	BIO 7	BIO 8	BIO 10	BIO 13	BIO 15	BIO 17
*M. montevidensis* (47)	0.997	0.350	44.7								26.1	8.3
*M. spectabilis* (5)	0.997	0.551		9.2							36.9	35.1
*M. stelzneri* (34)	0.997	0.151	20.3				33.8					10.7
*M.* sp.2 (5)	1	0.477	39.6		33							10.8
*M. cambaraensis* (3)	0.997	0.725	14.4								34.9	22.8
*M.* sp.3 (9)	0.982	0.506			70.6		9.3		6.7			
*M. tumifrons* (7)	0.991	0.356			22.6						48.9	13.5
*M. macrogranulosus* (2)	1	0.610		34.8	27						19.7	
*M. atroluteus* (70)	0.989	0.107				7.9	32.6					49.9
*M. sanmartini* (9)	0.982	0.416			38.5						50.3	4.7
*M. rubriventris* (14)	0.997	0.385	39.6				17.2			12.1		
*M*. sp.1 (9)	0.981	0.373			11.3						40.8	26.6
*M. simplex* (12)	0.997	0.438	17.4								28.8	21.1
*M. pachyrhynus* (14)	0.994	0.210			29.2				10.1		32.2	
*M. fulvoguttatus* (20)	0.980	0.152					24		16.2			37.9
*M. moreirae* (4)	0.992	0.317				30.3				44.6		16.3
*M. dorsalis* (20)	0.984	0.026	39.8		14							16.2
*M. langonei* (2)	0.997	0.540			37.5	19.2						19.6
*M. krauczuki* (8)	0.998	0.528			15.8	29.9						29.9
*M. estebani* (3)	0.966	0.575			11.3		76.5			5.6		
*M. devincenzii* (41)	0.991	0.180				3	35.1					56.5
*M. klappenbachi* (20)	0.990	0.087	19		22.8				25.9			
*M. paraguayensis* (10)	0.990	0.441		9.6	37.5				35.1			
*M. cupreuscapularis* (6)	0.999	0.329		12.2		39			39.2			

AUC mean and threshold values, and the percentage contribution of three most important bioclimatic variables to the distribution models for each *Melanophryniscus* species. See the meaning of bioclimatic variables in Dataset S4; threshold and AUC are explained in the Methods section.

The bioclimatic variables that most frequently presented a high contribution to the climatic suitability models were: Isothermality (the diurnal temperature range divided by the seasonal temperature range) (BIO 3), the Coefficient of Variation of Seasonal Precipitation (BIO 15), or the Precipitation of Driest Quarter (BIO 17). At least one of these was between the two most important bioclimatic variables for almost 80% of species (Table 1).

The current projected suitable areas varied from 720,505 km^2^, (for *Melanophryniscus klappenbachi*), to 4,461 km^2^ (for *M. macrogranulosus*) (Table 2). The projected range of suitable areas estimated for most species (n = 11) were less than 100,000 km^2^. In two species, the estimated range of suitable areas were smaller than 10,000 km^2^, and for five species they were between 10,000 and 50,000 km^2^ (Table 2).

**Table 2:** Summary of *Melanophryniscus* species data and results.

Species (n)	Present		Lost by 2020								Remaining by 2080				% area reduction by 2080
	Presence	Range area	Presence sites (n)			Range area (km^2^)			Presence sites (n)			Range area (km^2^)			
	sites (n)	(km^2^)	a2a	b2a	consensus	a2a	b2a	consensus	a2a	b2a	consensus	A2a	b2a	consensus	
*M. montevidensis* (47)	47	14148	47	47	47	14052	14140	14051	0	0	0	0	5.9	0	**100.0**
*M. spectabilis* (5)	5	74439	1	4	1	53197	56392	51825	3	1	1	11727	3067	1472	**90.1**
*M. stelzneri* (34)	34	58018	3	2	2	22219	15250	14754	18	31	18	16628	33.953	16547	**85.6**
*M.* sp.2 (5)	5	13202	4	4	4	10611	8355	8052	1	1	1	2531	4126	2146	**74.8**
*M. cambaraensis* (3)	3	7431	2	2	2	4580	4617	4277	3	2	1	2175	2766	1829	**66.8**
*M*. sp.3 (9)	8	461281	2	6	2	275918	306043	208374	6	3	3	167993	139334	73227	**66.7**
*M. tumifrons* (7)	7	225923	3	3	3	144426	137292	125071	4	4	4	79959	72230	65894	**66.3**
*M. macrogranulosus* (2)	2	4461	0	1	0	2410	2869	2279	2	1	1	2046	1591	1458	**59.2**
*M. atroluteus* (70)	68	469971	2	10	0	1.68	68067	1.68	5	53	3	140370	296423	129999	**53.5**
*M. sanmartini* (9)	9	393367	6	2	1	190300	169821	148521	3	3	3	202733	184850	174890	**50.7**
*M. rubriventris* (14)	13	72980	1	1	1	533	1511	451	12	13	12	20184	54111	20184	**49.1**
*M*. sp.1 (9)	8	593920	2	2	2	250311	222956	180373	7	7	7	325651	310734	278823	**46.4**
*M. simplex* (12)	11	75275	1	5	1	16301	41250	15821	11	7	7	58971	33831	33490	**38.4**
*M. pachyrhynus* (14)	14	184733	1	1	1	50545	70430	43885	13	13	13	134105	112981	106937	**33.1**
*M. fulvoguttatus* (20)	19	658511	1	1	1	97838	79047	64953	18	19	18	431161	485584	408698	**30.4**
*M. moreirae* (4)	4	35326	0	0	0	0	0	0	3	4	3	15896	35218	15896	**27.7**
*M. dorsalis* (20)	20	221801	1	1	1	37286	35728	24004	19	19	19	172063	184368	161003	**19.7**
*M. langonei* (2)	2	112571	0	0	0	5229	15167	5228	2	2	2	107342	97404	97404	**9.1**
*M. krauczuki* (8)	8	31461	0	0	0	3057	0	0	8	8	8	28404	31461	28404	**4.9**
*M. estebani* (3)	2	452948	0	1	0	24387	12939	11047	2	2	2	426.658	439.857	424766	**4.3**
*M. devincenzii* (41)	39	289317	1	1	0	11628	6590	3408	39	39	39	276871	282512	273766	**3.3**
*M. klappenbachi* (20)	19	720505	1	1	1	4701	736	487	19	19	19	714312	714987	709445	**0.8**
*M. paraguayensis* (10)	10	124696	0	0	0	32.13	0	0	10	10	10	124658	124695	124658	**0.0**
*M. cupreuscapularis* (6)	5	30356	0	0	0	0	0	0	5	5	5	30356	30356	30356	**0.0**

Estimation of percent reduction in the number of known presence sites and in the potential distribution area of *Melanophryniscus* species between present and 2080, considering two different climate scenarios (A2a and A2a), and a consensus between the scenarios.

### Potential magnitude of change in the size of species suitable areas

The estimated reduction in the geographic range of suitable conditions was widely variable at the species level and between the different scenarios (Table 2, Fig. S1–S6). Scenario A2a more frequently resulted in large decreases of suitable areas in 2080, with reductions of up to 70% for seven species. In Scenario B2a, a similar decrease in suitable climatic area in 2080 was predicted for only three species (Table 2, Fig. S1–S6), although nine species were predicted to have larger area reductions in comparison to Scenario A2a.

Considering the consensus scenario, 25% of species were predicted to have less than 10% reduction in their projected suitable areas, 40% were estimated to present more than 50% reduction. Particularly large losses of favorable habitat within projected suitable areas were projected for *Melanophryniscus spectabilis* and *M. montevidensis*, which were associated with estimated decreases of 90.1% and 100%, respectively (Table 2, Fig. S3 and S5). The projected range of suitable areas for 14 species was estimated to decrease up to 50% by the year 2080.

### Potential loss of known populations

The MaxEnt models for the year 2080 indicated that the climatic conditions at a number of known presence sites – here assumed to be different population units or subunits – may no longer be suitable for the persistence of certain species (Table 2). For four species a pronounced reduction was predicted, with more than 40% of known presence sites below the presence threshold already in 2020 (Table 2, Fig. S1-S6). Nevertheless, for most species (60%), all of the currently known occurrence sites remained above the presence thresholds in 2080. In general, those species with larger estimated percent loss in area also presented the largest percent loss in currently known presence sites (r^2^ = 0.91).

### Effects related to short-, and long-term climate change

We found that even in the short term models (2020) and regardless of the scenario considered, some species, like *Melanophryniscus montevidensis* and *M. stelzneri*, could already lose the totality of their projected suitable areas (Table 2, Fig. S1–S6). Furthermore, as previously mentioned, known populations of at least ten species (Fig. S1–S6) would fall in areas below the threshold of low climatic suitability. In contrast, three species (*M. klappenbachi*, *M. paraguayensis*, and *M. cupreuscapularis*) showed little or no reduction in their original projected suitable areas over the time period considered (Table 2, Figs. S1, S2, and S4).

### Correlations with original distribution range, phylogenetic groups and Major Habitat types

**Figure 2:**
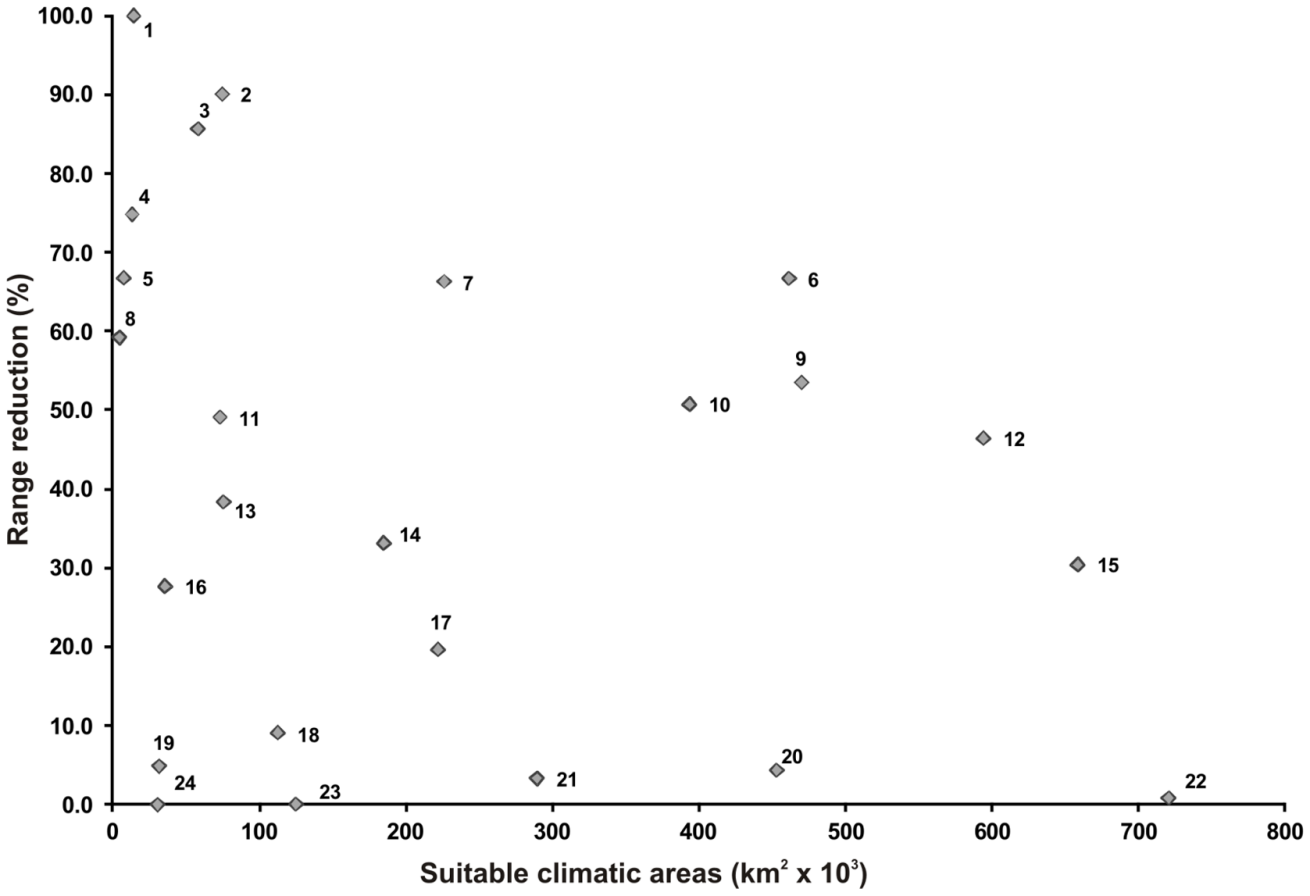
Relation between present size of species range and its percentage reduction in the future. Areas were estimated by MaxEnt models using bioclimatic variables; values are percentage reduction projected by 2080 (consensus between the IPCC A2a and B2a scenarios) for each species of *Melanophryniscus*. 1 - *M. montevidensis*, 2 - *M. spectabilis*, 3 - *M. stelzneri*, 4 - *M*. sp.2, 5 - *M. cambaraensis*, 6 - *M*. sp.3, 7 - *M. tumifrons*, 8 - *M. macrogranulosus*, 9 - *M.atroluteus*, 10 - *M. sanmartini*, 11 - *M. rubriventris*, 12 - *M*. sp.1, 13 - *M. simplex*, 14 - *M. pachyrhynus*, 15 - *M. fulvoguttatus*, 16 - *M. moreirae*, 17 - *M. dorsalis*, 18 - *M. langonei*, 19 - *M. krauczuki*, 20 - *M. estebani*, 21 - *M. devincenzii*, 22 - *M. klappenbachi*, 23 - *M. paraguayensis* and 24 - *M. cupreuscapularis*.

**Figure 3:**
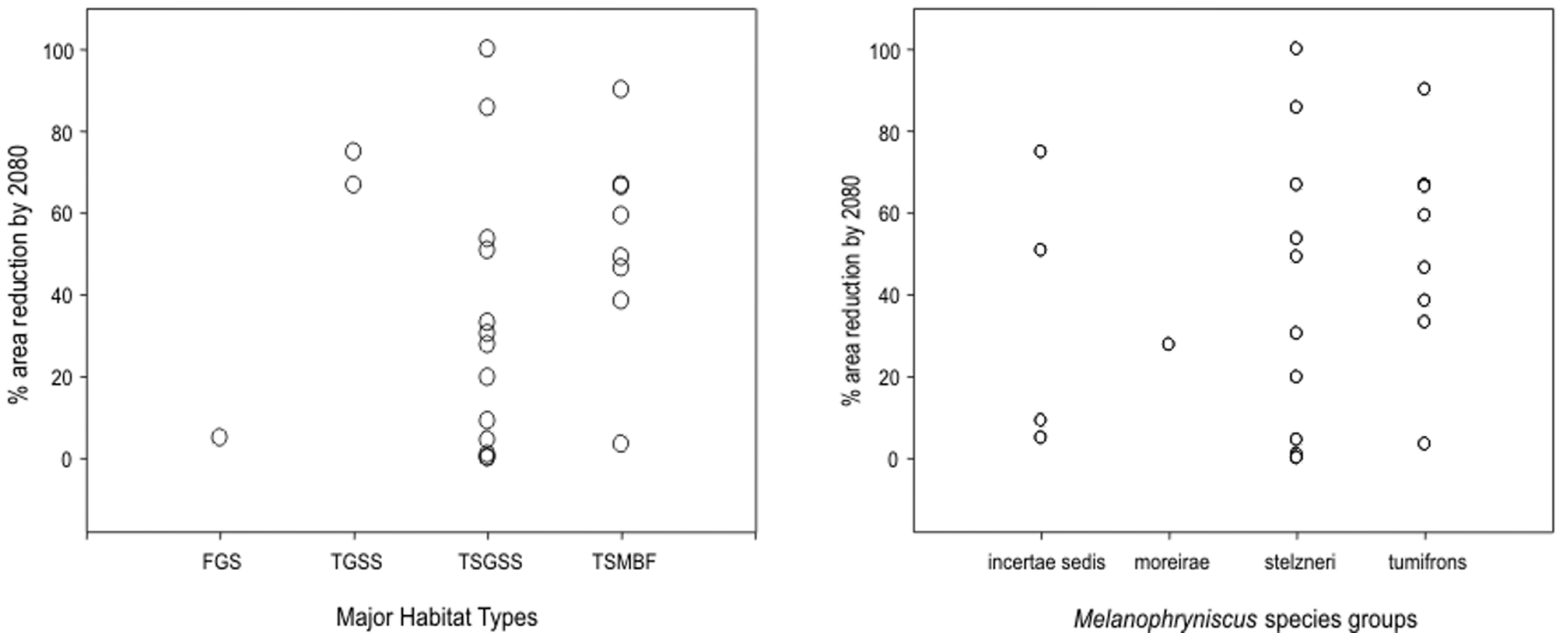
Reduction in climatic suitability area for *Melanophryniscus* species (2080) according to major habitat types in South America, and to phylogenetic groups. No significant differences were found in either situations (t-tests, TSGSS vs. TSMBF, p>0;19; stelzneri vs tumifrons, p>0.39). TSGSS, Tropical and Subtropical Grasslands, Savannas and Shrublands; TSMBF, Tropical and Subtropical Moist Broadleaf Forests.

Using a consensus of scenarios A2a and B2a, we found no significant correlation between the estimated area loss (%) by 2080 and the size of the original distribution range (Spearman rank order correlation, r_S_ = −0.314, p = 0.135). There was considerable variation across species in the magnitude of the projected area losses, especially for species with current suitable areas of less than 100,000 km^2^ (Fig. 2). We also found no significant differences in the estimated percent area lost by 2080 between species occurring along the main Major Habitat types (“Tropical and Subtropical Grasslands, Savannas and Shrublands” vs “Tropical and Subtropical Moist Broadleaf Forests”; t-test, p = 0.192), or between the two larger groups of species in *Melanophryniscus* (stelzneri and tumifrons, t-test, p = 0.398) (Fig. 3).

### Prioritization of species and populations for conservation

**Figure 4:**
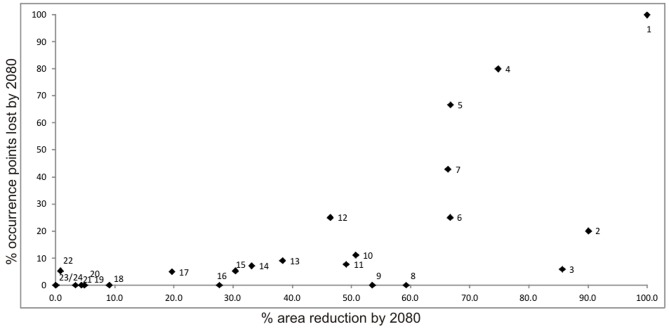
Scatterplot showing the relation between % reduction in known occurrence sites and % reduction in range area by 2080 (consensus scenario for IPCC A2A and B2A climate models). Conservation priority is high for species with high loss values measured in both % area and % occurrence points. 1 - *M. montevidensis*, 2 - *M. spectabilis*, 3 - *M. stelzneri*, 4 - *M*. sp.2, 5 - *M. cambaraensis*, 6 - *M*. sp.3, 7 - *M. tumifrons*, 8 - *M. macrogranulosus*, 9 - *M.atroluteus*, 10 - *M. sanmartini*, 11 - *M. rubriventris*, 12 - *M*. sp.1, 13 - *M. simplex*, 14 - *M. pachyrhynus*, 15 - *M. fulvoguttatus*, 16 - *M. moreirae*, 17 - *M. dorsalis*, 18 - *M. langonei*, 19 - *M. krauczuki*, 20 - *M. estebani*, 21 - *M. devincenzii*, 22 - *M. klappenbachi*, 23 - *M. paraguayensis* and 24 - *M. cupreuscapularis*.

Based on the percent decreases in the distribution areas projected for 2080, and the percent of known occurrence points potentially lost by 2080, four *Melanophryniscus* species, and also their populations, can be set to high priority for conservation and monitoring (Fig. 4): *M. montevidensis*, (with 100% of its original suitable range and all known occurrence points potentially lost by 2080), followed by *M*. sp.2, *M. cambaraensis* and *M. tumifrons*. Three other species (*M. spectabilis*, *M. stelzneri*, and *M*. sp.3) can be set between high and intermediate priority because they are predicted to lose climatic suitability in more than 60% of their present (∼2000) range, although the predicted loss of currently known occurrence points is below 30%. Nine other species can be ranked as intermediate priority, with predicted losses in area or known occurrence points between ca. 30% and 60%, while eight species can be ranked as low conservation priority in terms of global climatic change.

## Discussion

Our results indicate that species of *Melanophryniscus* may be affected by climate changes in different ways. The projected suitable areas of some species were drastically different from the present, while for other species only minor changes were predicted. As a consequence, conservation, research and monitoring efforts should be prioritized specifically for those species which are expected to be more readily and largely affected by climate change (see Fig. 2 and Table 2). Among the species for which marked reductions in projected suitable areas are expected by 2080, *M. montevidensis*, *M*. sp.2, and *M. cambaraensis* have particularly small range sizes, which might consequently make these species more sensitive to climate change if there are any concomitant pressure from other environmental alterations.

The results for species with less than 10 occurrence points, as *Melanophryniscus* sp.2, should be treated with caution because predictive power decreases with low sample sizes [58], [59]. Nevertheless, the low number of known occurrences for several *Melanophryniscus* species is a consequence of their naturally small geographical distributions and not of undersampling their total distribution. Therefore, adding new occurrences would only increase the density of records within the already sampled small area, and would not improve the performance of the models.

The genus *Melanophryniscus* includes a high number of species endangered at regional, national or global levels [19], [36], [37], [38], [39], [40], which are threatened by several factors apart from global climate change [19]. The fact that these species are already under extinction risk and usually have small distribution areas makes them even more vulnerable to extinction, since relatively small geographic shifts in climatic suitability could affect almost the whole range of a given species. Examples from the present study are the already endangered *M. montevidensis*, *M. cambaraensis*, and *M. macrogranulosus*, for which an area reduction of more than 50% was projected, should be given special attention. On the other hand, the models for at least three endangered species (*M. devincenzii*, *M. dorsalis*, and *M. langonei*) predicted small losses in area of suitable climatic conditions by 2080. This means that having a small range area is not sufficient to predict that a species will be more vulnerable to climatic changes and that the effects of climate change can be very different even among congener species, demanding different degrees of prioritization. For *Melanophryniscus*, the magnitude of potential change was not related to the size of the present suitable areas, *i.e*., species with more restricted ranges would not necessarily be more affected by climate change than the ones that are more broadly distributed. This result contrasts with the findings of a previous study [60] in which the range of current projected suitable areas was found to be inversely related to the projected area loss, indicating a disproportionately greater vulnerability for species with restricted distributions. In fact, a weak relation between species range sizes and climatic change is not surprising, since the spatial distribution of climatic changes is not homogeneous across the geographic space. In some regions, the climate does not change as much as in others, even within biomes, or the Major Habitat Types used in our analyses. This intra-regional heterogeneity in climatic change is implicit in Figure 3, where the predicted area reduction within each Major Habitat Type (e.g., TSGSS) varied from no change (0%) to high changes (ca. 100%) in area. Therefore, species with small ranges will only be affected by climate change if their ranges are geographically coincident with the regions suffering major climatic changes, e.g., species 1 to 5 in Fig. 2 (see also Figs. S1 to S6). On the other hand, when there is no such coincidence, species with small ranges would not be affected by climatic changes (e.g., species 18,19, 23, 24 in Fig. 2).

By examining the projected changes in the climatic suitability for known populations of each species of *Melanophryniscus*, we identified two contrasting groups of populations (i.e., each known presence site was considered to be a population). The first group includes populations for which the area of suitable climatic conditions was not expected to decrease by the year 2080 (see Figs. S1 to S6). The second group includes populations projected to lose areas with suitable climatic conditions already by the year 2020. Monitoring this second group of populations to document trends in population sizes, reproductive events, and local habitat conditions (especially those that can be related to climate, distribution, amount and duration of local rains), would provide empirical data to test the accuracy of our projections. Data on these populations should also prove useful in supporting the development of more effective conservation plans for these species. Additionally, because these populations are expected to be the first *Melanophryniscus* populations to be affected by climate change, they are the most suited targets for *in situ* conservation efforts, such as breeding site enhancement and manipulation of the hydroperiod or water levels at breeding sites [61].

We consider *in situ* studies, particularly those involving long-term monitoring of known populations, to be a necessary approach for testing the accuracy of our model projections. In the absence of better ecological information, the bioclimatic variables that contribute most in generating the projected suitable areas models for each species may be used to select which local environmental conditions we should monitor in the future (Table 1). For instance, BIO 17 (precipitation of the driest quarter) and BIO 15 (coefficient of variation of seasonal precipitation) were important in the models of several species, suggesting that local precipitation patterns should be monitored. This is consistent with the life-history characteristics of *Melanophryniscus*, since their reproductive events are known to be related to intense rainfall [33], [34], [35]. Additionally, it would be important to investigate what are the thresholds of rainfall that trigger the reproductive events. Therefore, monitoring of local climatic conditions and their relation with *Melanophryniscus* population dynamics could be informative of thresholds and habitat requirements useful in conservation actions.

The usefulness of models of suitable climatic areas in helping to set conservation strategies for several taxa is undisputed (e.g. [12], [62], [63], [64], [65]). According to our results for *Melanophryniscus* toads (and probably also for other species with restricted ranges and limited dispersal ability), comparing modeled areas of present and future climatic suitability seems a useful approach for determining which species might suffer earlier reductions in habitat availability. Clearly, these species should be set as priorities for research and conservation, although we recognize that climate change analyses contain uncertainties [66], [67] and that climate change is not the only or even the main factor threatening all amphibian species (see [68], [69], [70], [71]).

In this study we predicted which *Melanophryniscus* species should be most vulnerable to climate change, based on the projected reduction in their suitable climatic areas. At the intra-specific level, analyzing the average percent reduction in the number of known presence sites (here assumed to be different populations), along with the percent reduction in potential ranges, provides an additional estimate of vulnerability for a given species. This analysis may be more useful to objectively pin-point priority conservation sites and to establish local level research, monitoring and conservation actions. In fact, by combining empirical data (analyses based on occurrence points) with projections generated by distribution models (analyses of projected suitable climatic areas), we here suggest one possible way of increasing the comprehensiveness, reliability and applicability of the assessments of climate change impacts.

## Supporting Information

Figure S1 **Modeled distribution maps.** Maps for *M. atroluteus*, *M. cambaraensis*, *M. cupreuscapularis*, and *M. devincenzii*. The maps show the potential distribution areas in present time (∼2000), the areas potentially lost by 2020, potentially remaining by 2020 and potentially remaining by 2080. The 2080 consensus of remaining areas represents regions that persist as climatically suitable in either scenarios A2a (left) and B2a (right).
(TIF)

Figure S2 **Modeled distribution maps.** Maps for *M. dorsalis*, *M. estebani*, *M. fulvoguttatus*, and *M. klappenbachi*. The maps show the potential distribution areas in present time (∼2000), the areas potentially lost by 2020, potentially remaining by 2020 and potentially remaining by 2080. The 2080 consensus of remaining areas represents regions that persist as climatically suitable in either scenarios A2a (left) and B2a (right).
(TIF)

Figure S3 **Modeled distribution maps.** Maps for *M. krauczuki*, *M. langonei*, *M. macrogranulosus*, and *M. montevidensis*. The maps show the potential distribution areas in present time (∼2000), the areas potentially lost by 2020, potentially remaining by 2020 and potentially remaining by 2080. The 2080 consensus of remaining areas represents regions that persist as climatically suitable in either scenarios A2a (left) and B2a (right).
(TIF)

Figure S4 **Modeled distribution maps.** Maps for *M. moreirae*, *M. pachyrhynus*, *M. paraguayensis*, and *M. rubriventris*. The maps show the potential distribution areas in present time (∼2000), the areas potentially lost by 2020, potentially remaining by 2020 and potentially remaining by 2080. The 2080 consensus of remaining areas represents regions that persist as climatically suitable in either scenarios A2a (left) and B2a (right).
(TIF)

Figure S5 **Modeled distribution maps.** Maps for *M. sanmartini*, *M. simplex*, *M. spectabilis*, and *M. stelzneri*. The maps show the potential distribution areas in present time (∼2000), the areas potentially lost by 2020, potentially remaining by 2020 and potentially remaining by 2080. The 2080 consensus of remaining areas represents regions that persist as climatically suitable in either scenarios A2a (left) and B2a (right).
(TIF)

Figure S6 **Modeled distribution maps.** Maps for *M. tumifrons*, *M*. sp.n.1, *M*. sp.n.2, and *M*. sp.n.3. The maps show the potential distribution areas in present time (∼2000), the areas potentially lost by 2020, potentially remaining by 2020 and potentially remaining by 2080. The 2080 consensus of remaining areas represents regions that persist as climatically suitable in either scenarios A2a (left) and B2a (right).
(TIF)

Dataset S1 ***Melanophryniscus*** **species.** Species of the genus *Melanophryniscus* included in this study.
(DOC)

Dataset S2 **Literature used.** The published literature used to obtain the presence locations for each study species: Caramaschi and Cruz, 2002; Baldo and Basso, 2004; Kwet et al., 2005; Brusquetti et al., 2007; Weber et al., 2007; Colombo et al., 2007; Langone et al., 2008; Maneyro and Kwet, 2008; Airaldi et al., 2009; Boeris et al., 2010; Bidau et al., 2011.
(DOC)

Dataset S3 **Scientific collections.** List of scientific collections containing *Melanophryniscus* samples reviewed for record and species validation.
(DOC)

Dataset S4 **Environmental variable.** List of variables used to model the potential distributions range of *Melanophryniscus* species. Source: project Worldclim versão 1.4 (http://www.worldclim.org).
(DOC)

Dataset S5 ***Melanophryniscus*** **species and its major habitat type and phylogenetic groups.** *Melanophryniscus* species: major habitat types in South America (those including more than 50% of each species distribution), and phylogenetic groups. Major Habitat Types (Olson, 2001): TSGSS  =  Tropical and Subtropical Grasslands, Savannas and Shrublands, TSMBF  =  Tropical and Subtropical Moist Broadleaf Forests, TGSS  =  Temperate Grasslands, Savannas and Shrublands. The taxonomic groups were defined based on Cruz and Caramaschi 2003; Baldo *et al*., 2012, and Baldo *et al*., unpubl. data.
(DOC)
