# Supplementary figures and images for: Climate Change and the Distribution of Neotropical Red-Bellied Toads (Melanophryniscus, Anura, Amphibia): How to Prioritize Species and Populations?
Source: PLoS One. 2014 Apr 22;9(4):e94625. doi: 10.1371/journal.pone.0094625 (PMC3995645; doi:10.1371/journal.pone.0094625)

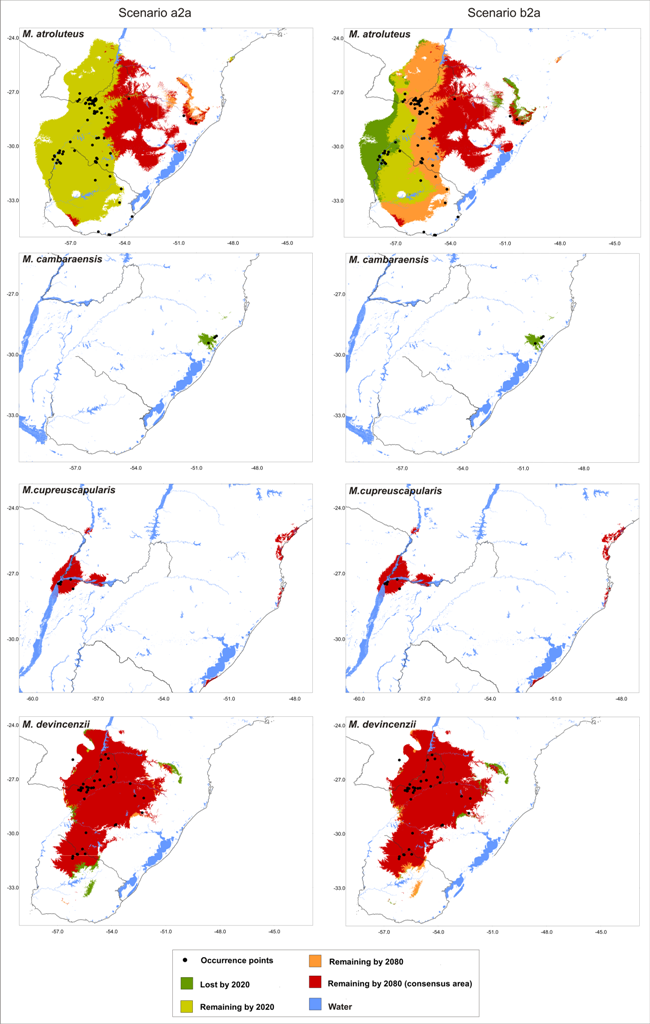

Supplement: Figure S1 — Modeled distribution maps. Maps for M. atroluteus, M. cambaraensis, M. cupreuscapularis, and M. devincenzii. The maps show the potential distribution areas in present time (∼2000), the areas potentially lost by 2020, potentially remaining by 2020 and potentially remaining by 2080. The 2080 consensus of remaining areas represents regions that persist as climatically suitable in either scenarios A2a (left) and B2a (right). (TIF) [file pone.0094625.s001.tif]

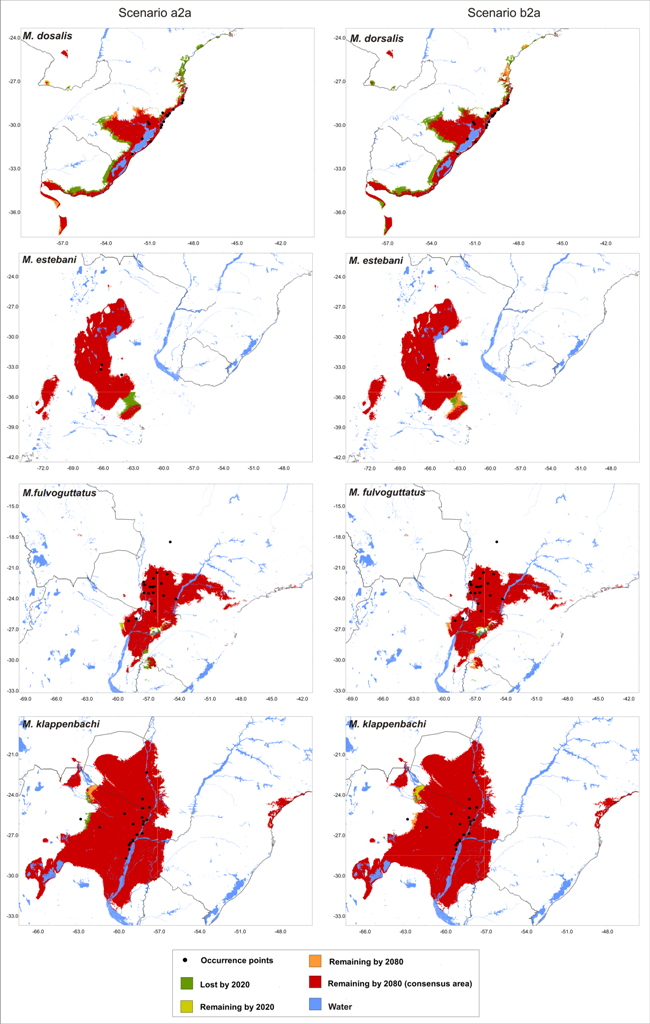

Supplement: Figure S2 — Modeled distribution maps. Maps for M. dorsalis, M. estebani, M. fulvoguttatus, and M. klappenbachi. The maps show the potential distribution areas in present time (∼2000), the areas potentially lost by 2020, potentially remaining by 2020 and potentially remaining by 2080. The 2080 consensus of remaining areas represents regions that persist as climatically suitable in either scenarios A2a (left) and B2a (right). (TIF) [file pone.0094625.s002.tif]

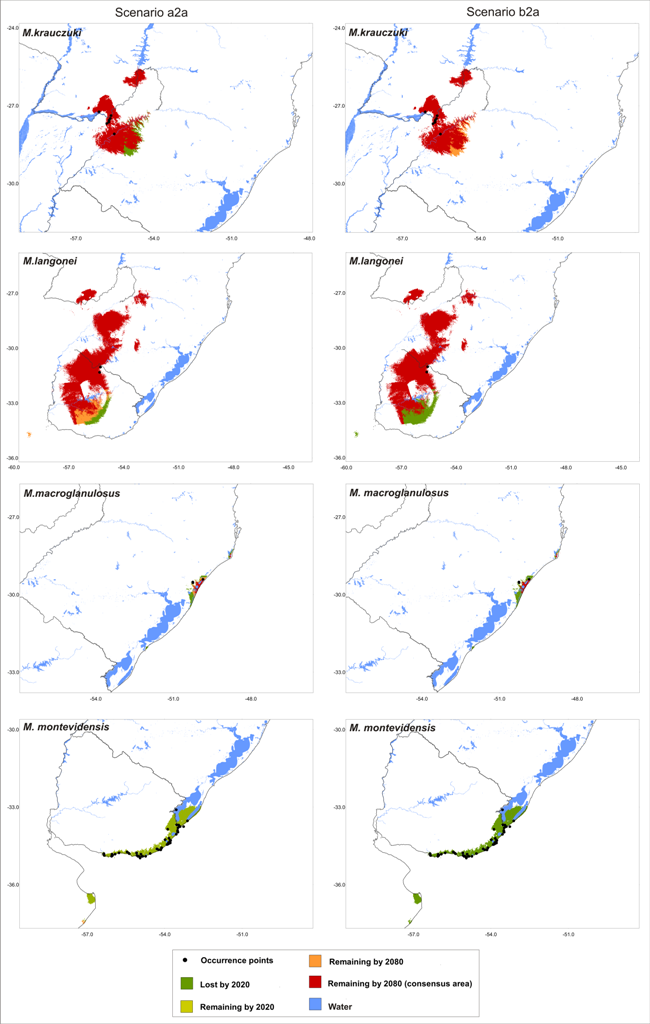

Supplement: Figure S3 — Modeled distribution maps. Maps for M. krauczuki, M. langonei, M. macrogranulosus, and M. montevidensis. The maps show the potential distribution areas in present time (∼2000), the areas potentially lost by 2020, potentially remaining by 2020 and potentially remaining by 2080. The 2080 consensus of remaining areas represents regions that persist as climatically suitable in either scenarios A2a (left) and B2a (right). (TIF) [file pone.0094625.s003.tif]

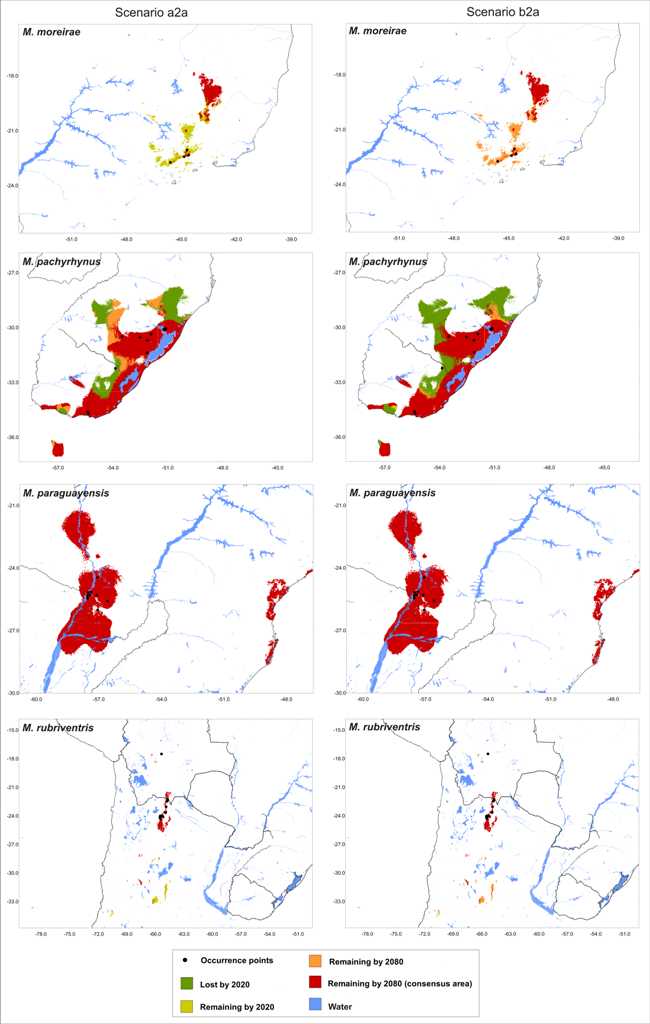

Supplement: Figure S4 — Modeled distribution maps. Maps for M. moreirae, M. pachyrhynus, M. paraguayensis, and M. rubriventris. The maps show the potential distribution areas in present time (∼2000), the areas potentially lost by 2020, potentially remaining by 2020 and potentially remaining by 2080. The 2080 consensus of remaining areas represents regions that persist as climatically suitable in either scenarios A2a (left) and B2a (right). (TIF) [file pone.0094625.s004.tif]

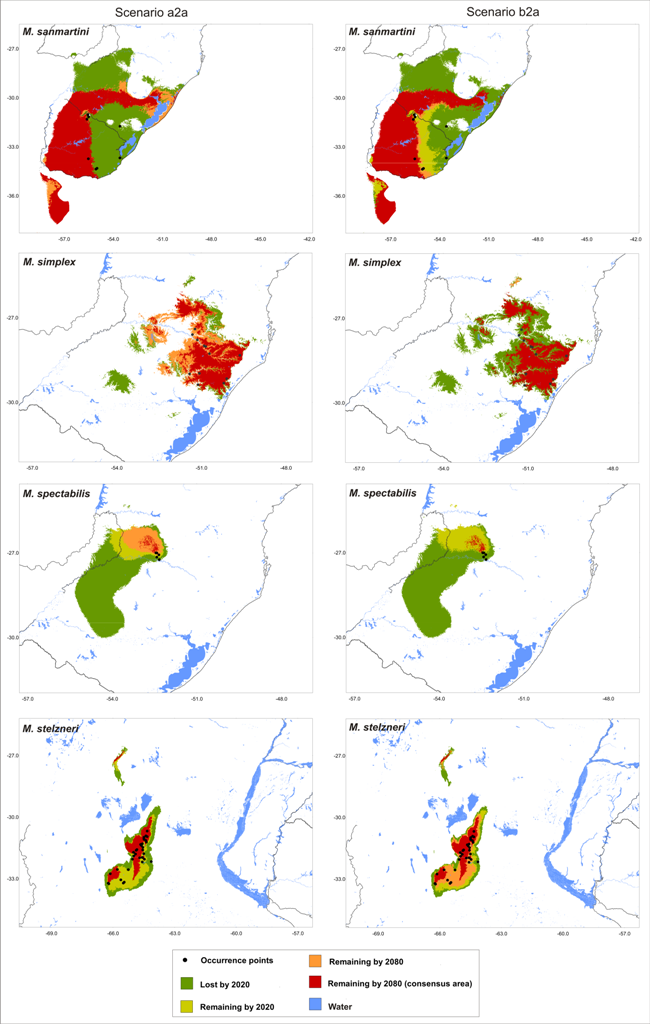

Supplement: Figure S5 — Modeled distribution maps. Maps for M. sanmartini, M. simplex, M. spectabilis, and M. stelzneri. The maps show the potential distribution areas in present time (∼2000), the areas potentially lost by 2020, potentially remaining by 2020 and potentially remaining by 2080. The 2080 consensus of remaining areas represents regions that persist as climatically suitable in either scenarios A2a (left) and B2a (right). (TIF) [file pone.0094625.s005.tif]

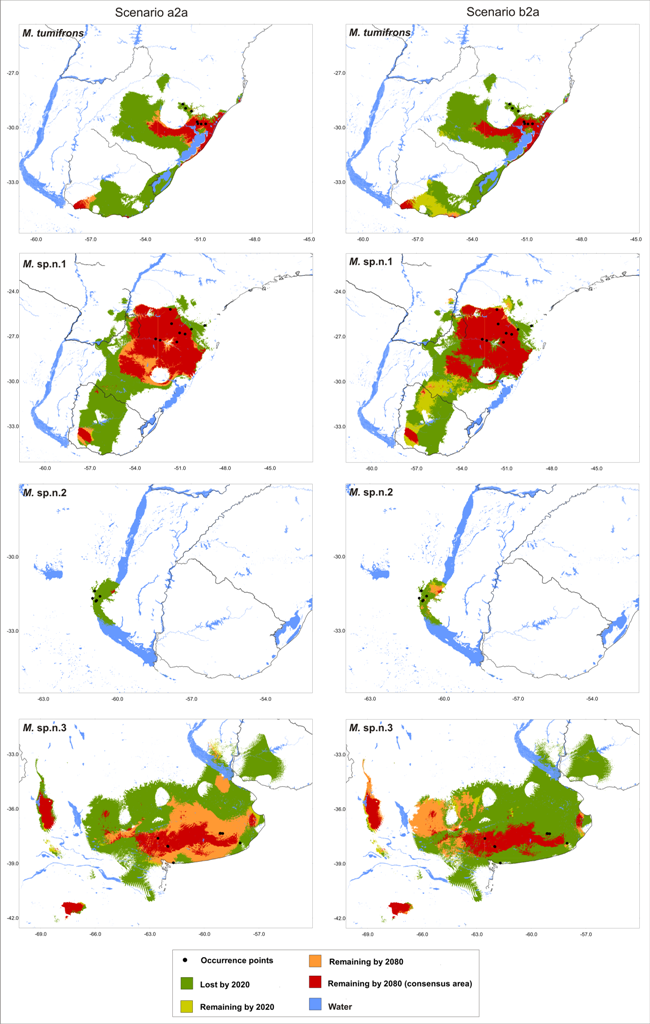

Supplement: Figure S6 — Modeled distribution maps. Maps for M. tumifrons, M. sp.n.1, M. sp.n.2, and M. sp.n.3. The maps show the potential distribution areas in present time (∼2000), the areas potentially lost by 2020, potentially remaining by 2020 and potentially remaining by 2080. The 2080 consensus of remaining areas represents regions that persist as climatically suitable in either scenarios A2a (left) and B2a (right). (TIF) [file pone.0094625.s006.tif]
