# Supplementary material for: Climate Change and the Distribution of Neotropical Red-Bellied Toads (Melanophryniscus, Anura, Amphibia): How to Prioritize Species and Populations?
Source: PLoS One. 2014 Apr 22;9(4):e94625. doi: 10.1371/journal.pone.0094625 (PMC3995645; doi:10.1371/journal.pone.0094625)
Supplement: Dataset S1 — Melanophryniscus species. Species of the genus Melanophryniscus included in this study. (DOC) [file pone.0094625.s007.doc]

**Dataset S1**

[*Melanophryniscus atroluteus*](http://research.amnh.org/vz/herpetology/amphibia/references.php?id=2961) ([Miranda-Ribeiro](http://research.amnh.org/vz/herpetology/amphibia/names.php?a_id=51), [1920](http://research.amnh.org/vz/herpetology/amphibia/names.php?year=1920)), [*Melanophryniscus cambaraensis*](http://research.amnh.org/vz/herpetology/amphibia/references.php?id=2965) [Braun and Braun](http://research.amnh.org/vz/herpetology/amphibia/names.php?a_id=540), [1979](http://research.amnh.org/vz/herpetology/amphibia/names.php?year=1979), [*Melanophryniscus cupreuscapularis*](http://research.amnh.org/vz/herpetology/amphibia/references.php?id=2966) [Céspedez and Alvarez](http://research.amnh.org/vz/herpetology/amphibia/names.php?a_id=541), [2000](http://research.amnh.org/vz/herpetology/amphibia/names.php?year=2000), [*Melanophryniscus devincenzii*](http://research.amnh.org/vz/herpetology/amphibia/references.php?id=2967) [Klappenbach](http://research.amnh.org/vz/herpetology/amphibia/names.php?a_id=542), [1968](http://research.amnh.org/vz/herpetology/amphibia/names.php?year=1968), [*Melanophryniscus dorsalis*](http://research.amnh.org/vz/herpetology/amphibia/references.php?id=2968) ([Mertens](http://research.amnh.org/vz/herpetology/amphibia/names.php?a_id=218), [1933](http://research.amnh.org/vz/herpetology/amphibia/names.php?year=1933)), *Melanophryniscus* *estebani* Céspedez, 2008, [*Melanophryniscus fulvoguttatus*](http://research.amnh.org/vz/herpetology/amphibia/references.php?id=2972) ([Mertens](http://research.amnh.org/vz/herpetology/amphibia/names.php?a_id=218), [1937](http://research.amnh.org/vz/herpetology/amphibia/names.php?year=1937)), [*Melanophryniscus klappenbachi*](http://research.amnh.org/vz/herpetology/amphibia/references.php?id=2976) [Prigioni and Langone](http://research.amnh.org/vz/herpetology/amphibia/names.php?a_id=544), [2000](http://research.amnh.org/vz/herpetology/amphibia/names.php?year=2000), [*Melanophryniscus krauczuki*](http://research.amnh.org/vz/herpetology/amphibia/references.php?id=2977) [Baldo and Basso](http://research.amnh.org/vz/herpetology/amphibia/names.php?a_id=545), [2004](http://research.amnh.org/vz/herpetology/amphibia/names.php?year=2004), [*Melanophryniscus langonei*](http://research.amnh.org/vz/herpetology/amphibia/references.php?id=2978) [Maneyro, Naya, and Baldo](http://research.amnh.org/vz/herpetology/amphibia/names.php?a_id=546), [2008](http://research.amnh.org/vz/herpetology/amphibia/names.php?year=2008), [*Melanophryniscus macrogranulosus*](http://research.amnh.org/vz/herpetology/amphibia/references.php?id=2979) [Braun](http://research.amnh.org/vz/herpetology/amphibia/names.php?a_id=547), [1973](http://research.amnh.org/vz/herpetology/amphibia/names.php?year=1973), [*Melanophryniscus montevidensis*](http://research.amnh.org/vz/herpetology/amphibia/references.php?id=2980) ([Philippi](http://research.amnh.org/vz/herpetology/amphibia/names.php?a_id=76), [1902](http://research.amnh.org/vz/herpetology/amphibia/names.php?year=1902)), [*Melanophryniscus moreirae*](http://research.amnh.org/vz/herpetology/amphibia/references.php?id=2984) ([Miranda-Ribeiro](http://research.amnh.org/vz/herpetology/amphibia/names.php?a_id=51), [1920](http://research.amnh.org/vz/herpetology/amphibia/names.php?year=1920)), [*Melanophryniscus pachyrhynus*](http://research.amnh.org/vz/herpetology/amphibia/references.php?id=2992) ([Miranda-Ribeiro](http://research.amnh.org/vz/herpetology/amphibia/names.php?a_id=51), [1920](http://research.amnh.org/vz/herpetology/amphibia/names.php?year=1920)), [*Melanophryniscus paraguayensis*](http://research.amnh.org/vz/herpetology/amphibia/references.php?id=2995) [Céspedez and Motte](http://research.amnh.org/vz/herpetology/amphibia/names.php?a_id=550), [2007](http://research.amnh.org/vz/herpetology/amphibia/names.php?year=2007), [*Melanophryniscus rubriventris*](http://research.amnh.org/vz/herpetology/amphibia/references.php?id=2996) ([Vellard](http://research.amnh.org/vz/herpetology/amphibia/names.php?a_id=551), [1947](http://research.amnh.org/vz/herpetology/amphibia/names.php?year=1947)), [*Melanophryniscus sanmartini*](http://research.amnh.org/vz/herpetology/amphibia/references.php?id=3003) [Klappenbach](http://research.amnh.org/vz/herpetology/amphibia/names.php?a_id=542), [1968](http://research.amnh.org/vz/herpetology/amphibia/names.php?year=1968), [*Melanophryniscus simplex*](http://research.amnh.org/vz/herpetology/amphibia/references.php?id=3004) [Caramaschi and Cruz](http://research.amnh.org/vz/herpetology/amphibia/names.php?a_id=549), [2002](http://research.amnh.org/vz/herpetology/amphibia/names.php?year=2002), [*Melanophryniscus spectabilis*](http://research.amnh.org/vz/herpetology/amphibia/references.php?id=3005) [Caramaschi and Cruz](http://research.amnh.org/vz/herpetology/amphibia/names.php?a_id=549), [2002](http://research.amnh.org/vz/herpetology/amphibia/names.php?year=2002), [*Melanophryniscus stelzneri*](http://research.amnh.org/vz/herpetology/amphibia/references.php?id=3006) ([Weyenbergh](http://research.amnh.org/vz/herpetology/amphibia/names.php?a_id=553), [1875](http://research.amnh.org/vz/herpetology/amphibia/names.php?year=1875)) and [*Melanophryniscus tumifrons*](http://research.amnh.org/vz/herpetology/amphibia/references.php?id=3013) ([Boulenger](http://research.amnh.org/vz/herpetology/amphibia/names.php?a_id=26), [1905](http://research.amnh.org/vz/herpetology/amphibia/names.php?year=1905)). Additionally, we use three new taxon not yet described for the genus (Diego Baldo, Personal observation), called here as *Melanophryniscus* sp.1, *Melanophryniscus* sp.2 and *Melanophryniscus* sp.3.
