# Supplementary material for: Climate Change and the Distribution of Neotropical Red-Bellied Toads (Melanophryniscus, Anura, Amphibia): How to Prioritize Species and Populations?
Source: PLoS One. 2014 Apr 22;9(4):e94625. doi: 10.1371/journal.pone.0094625 (PMC3995645; doi:10.1371/journal.pone.0094625)
Supplement: Dataset S2 — Literature used. The published literature used to obtain the presence locations for each study species: Caramaschi and Cruz, 2002; Baldo and Basso, 2004; Kwet et al., 2005; Brusquetti et al., 2007; Weber et al., 2007; Colombo et al., 2007; Langone et al., 2008; Maneyro and Kwet, 2008; Airaldi et al., 2009; Boeris et al., 2010; Bidau et al., 2011. (DOC) [file pone.0094625.s008.doc]

**Dataset S2**

References:

Airaldi K, Baldo D, Lavilla EO (2009) Amphibia, Anura, Bufonidae, *Melanophryniscus devincenzii*: First record for Paraguay and geographic distribution map. Check List 5: 377-379.

Baldo D, Basso NG (2004) A New Species of *Melanophryniscus* Gallardo, 1961 (Anura: Bufonidae), with Comments on the Species of the Genus Reported for Misiones, Northeastern Argentina. J Herpetol 38: 393-403.

Bidau CJ, Martí DA, Baldo D (2011) Inter- and Intraspecific Geographic Variation of Body Size in South American Redbelly Toads of the Genus *Melanophryniscus* Gallardo, 1961 (Anura: Bufonidae). J Herpetol 45: 66-74.

Boeris J, Ferro MJ, Krauczuk E, Baldo D (20100 Amphibia, Anura, Bufonidae, *Melanophryniscus devincenzii* Klappenbach, 1968: First record for Corrientes Province, Argentina. Check List 6: 395-396.

Brusquetti F, Baldo D, Motte M (2007) Amphibia, Anura, Bufonidae, *Melanophryniscus krauczuki*: Geographic distribution map and the first record for Paraguay. Check List 3: 141-142.

Caramaschi U, Cruz CAG (2002) Taxonomic status of *Atelopus pachyrhynus* Miranda-Ribeiro, 1920, redescription of *Melanophryniscus tumifrons* (Boulenger, 1905), and descriptions of two new species of *Melanophryniscus* from the state of Santa Catarina, Brazil (Amphibia, Anura, Bufonidae). Arq Mus Nac 60: 303-314.

Colombo P, Zank C, Schmidt LES, Gonçalves G, Marinho JR (2007) Anura, Bufonidae, *Melanophryniscus simplex*: Distribution extension. Check List 3: 305-307.

De La Riva I (1995) *Melanophryniscus rubriventris*. Herpetol Rev 26: 152-153.

Kwet A, Maneyro R, Zillikens A, Mebs D (2005) Advertisement calls of *Melanophryniscus dorsalis* ( Mertens , 1933 ) and *M . montevidensis* ( Philippi, 1902 ), two parapatric species from southern Brazil and Uruguay, with comments on morphological variation in the *Melanophryniscus stelzneri* group (Anura: Bufonidae). Salamandra 41: 1-18.

Langone JA, Segalla MV, Bornschein M, Sá ROD (2008) A new reproductive mode in the genus *Melanophryniscus* Gallardo , 1961 (Anura: Bufonidae ) with description of a new species from the state of Paraná, Brazil. South Am J Herpetol 3: 1-9.

Maneyro R, Kwet A (2008) Amphibians in the border region between Uruguay and Brazil : Updated species list with comments on taxonomy and natural history (Part I : Bufonidae ). Palaeodiversity 1: 95-121.

Weber LN, Procaci LS, Salles ROL, Silva SP, Corrêa AL et al. (2007) Amphibia, Anura, Bufonidae, *Melanophryniscus moreirae*: Distribution extension. Check List 3: 346-347.
