# Supplementary material for: Climate Change and the Distribution of Neotropical Red-Bellied Toads (Melanophryniscus, Anura, Amphibia): How to Prioritize Species and Populations?
Source: PLoS One. 2014 Apr 22;9(4):e94625. doi: 10.1371/journal.pone.0094625 (PMC3995645; doi:10.1371/journal.pone.0094625)
Supplement: Dataset S3 — Scientific collections. List of scientific collections containing Melanophryniscus samples reviewed for record and species validation. (DOC) [file pone.0094625.s009.doc]

**Dataset S3**

**Brazil:** Coleção de Anfíbios Universidade de Passo Fundo (CAUPF), Museu de Ciências Naturais da Fundação Zoobotânica do Rio Grande do Sul (MCN), Museu de Ciências e Tecnologia da Pontifícia Universidade Católica do Rio Grande do Sul (MCP), Coleção de Anfíbios da Universidade Federal do Rio Grande do Sul (UFRGS), Coleção de Anfíbios da Universidade Federal de Santa Maria (ZUFSM), Museu Nacional do Rio de Janeiro (MNRJ), Coleção Adolpho Lutz no MNRJ (AL-MN), Museu de Zoologia da Universidade de São Paulo (MZUSP), Coleção Célio Fernando Baptista Haddad (CFBH). **Argentina:** Museo Argentino de Ciencias Naturales Bernardino Rivadavia (MACN), Fundación Miguel Lillo - Instituto de Herpetología, (FML), Universidad Nacional de Misiones (CHUNAM), Museo Argentino Ameghino (MFA-ZV.H), Museo de Zoología, Universidad Nacional de Córdoba (MZUC), Colección Herpetológica Museo La Plata (MLP) and Museo de Ciencias Naturales, Universidad Nacional de Salta (MCN-UNSA). **Uruguay:** Museo Nacional de Historia Natural (MNHN) and Colección de Vertebrados, Facultad de Ciencias, UDELAR (ZVCB). **Paraguay:** Colección. Zoológica de la Facultad de Ciencias Exactas y Naturales (CZCEN) and Instituto de Investigación Biológica del Paraguay (IIBP). **Germany:** Staatlichen Museums für Naturkunde Stuttgart (SMNS). **France:** Museum National d'Histoire Naturelle d e Paris (MNHNP).
