# Supplementary material for: Climate Change and the Distribution of Neotropical Red-Bellied Toads (Melanophryniscus, Anura, Amphibia): How to Prioritize Species and Populations?
Source: PLoS One. 2014 Apr 22;9(4):e94625. doi: 10.1371/journal.pone.0094625 (PMC3995645; doi:10.1371/journal.pone.0094625)
Supplement: Dataset S4 — Environmental variable. List of variables used to model the potential distributions range of Melanophryniscus species. Source: project Worldclim versão 1.4 (http://www.worldclim.org). (DOC) [file pone.0094625.s010.doc]

**Dataset S4**

| **Variable Type** | **Variable Code** |
| --- | --- |
| Mean Diurnal Range: Mean of monthly (max temp - min temp) | BIO2 |
| Isothermality: (P2/P7)* 100 | BIO3 |
| Max Temperature of Warmest Month | BIO5 |
| Temperature Annual Range (P5-P6) | BIO7 |
| Mean Temperature of Wettest Quarter | BIO8 |
| Mean Temperature of Warmest Quarter | BIO10 |
| Precipitation of Wettest Month | BIO13 |
| Precipitation Seasonality (Coefficient of Variation) | BIO15 |
| Precipitation of Driest Quarter | BIO17 |
| Altitude | alt |
