# Supplementary material for: Climate Change and the Distribution of Neotropical Red-Bellied Toads (Melanophryniscus, Anura, Amphibia): How to Prioritize Species and Populations?
Source: PLoS One. 2014 Apr 22;9(4):e94625. doi: 10.1371/journal.pone.0094625 (PMC3995645; doi:10.1371/journal.pone.0094625)
Supplement: Dataset S5 — Melanophryniscus species and its major habitat type and phylogenetic groups. Melanophryniscus species: major habitat types in South America (those including more than 50% of each species distribution), and phylogenetic groups. Major Habitat Types (Olson, 2001): TSGSS = Tropical and Subtropical Grasslands, Savannas and Shrublands, TSMBF = Tropical and Subtropical Moist Broadleaf Forests, TGSS = Temperate Grasslands, Savannas and Shrublands. The taxonomic groups were defined based on Cruz and Caramaschi 2003; Baldo et al., 2012, and Baldo et al., unpubl. data. (DOC) [file pone.0094625.s011.doc]

**Dataset S5**

| **Species** | **Major Habitat Type** | **Taxonomic group** |
| --- | --- | --- |
| *M. montevidensis* (47) | TSGSS | stelzneri |
| *M. spectabilis* (5) | TSMBF | tumifrons |
| *M. stelzneri* (34) | TSGSS | stelzneri |
| *M.* sp.2 (5) | TGSS | indefinite |
| *M. cambaraensis* (3) | TSMBF | tumifrons |
| *M.* sp.3 (9) | TGSS | stelzneri |
| *M. tumifrons* (7) | TSMBF | tumifrons |
| *M. macrogranulosus* (2) | TSMBF | tumifrons |
| *M. atroluteus* (70) | TSGSS | stelzneri |
| *M. sanmartini* (9) | TSGSS | indefinite |
| *M. rubriventris* (14) | TSMBF | stelzneri |
| *M.* sp.1 (9) | TSMBF | tumifrons |
| *M. simplex* (12) | TSMBF | tumifrons |
| *M. pachyrhynus* (14) | TSGSS | tumifrons |
| *M. fulvoguttatus* (20) | TSGSS | stelzneri |
| *M. moreirae* (4) | TSGSS | moreirae |
| *M. dorsalis* (20) | TSGSS | stelzneri |
| *M. langonei* (2) | TSGSS | indefinite |
| *M. krauczuki* (8) | TSMBF | indefinite |
| *M. estebani* (3) | TSGSS | stelzneri |
| *M. devincenzii* (41) | TSMBF | tumifrons |
| *M. klappenbachi* (20) | TSGSS | stelzneri |
| *M. paraguayensis* (10) | TSGSS | stelzneri |
| *M. cupreuscapularis* (6) | TSGSS | stelzneri |
